# The addition of radiotherapy to breast-conserving surgery improves survival for elderly patients with early breast cancer

**DOI:** 10.3389/fonc.2022.917054

**Published:** 2022-11-23

**Authors:** Shi-Ping Yang, Lu-Lu Tan, Ping Zhou, Chen-Lu Lian, San-Gang Wu, Zhen-Yu He

**Affiliations:** ^1^ Department of Radiation Oncology, Hainan General Hospital, Hainan Affiliated Hospital of Hainan Medical University, Haikou, China; ^2^ Department of Radiation Oncology, Xiamen Cancer Center, Xiamen Key Laboratory of Radiation Oncology, The First Affiliated Hospital of Xiamen University, School of Medicine, Xiamen University, Xiamen, China; ^3^ Department of Radiation Oncology, Sun Yat-sen University Cancer Center, State Key Laboratory of Oncology in South China, Collaborative Innovation Center of Cancer Medicine, Guangzhou, China

**Keywords:** breast cancer, elderly, radiotherapy, breast-conserving surgery, SEER

## Abstract

**Purpose:**

To evaluate whether adjuvant radiotherapy (RT) after breast-conserving surgery (BCS) was associated with better survival among elderly (≥70 years) breast cancer patients with T1-2N0 and estrogen receptor (ER) positive disease.

**Methods:**

We included patients who met the inclusion criteria between 2010 and 2014 from the Surveillance, Epidemiology, and End Results program. Patients were subdivided into three groups based on surgery and RT: BCS alone, BCS plus RT, and refusal of RT. The primary outcomes were breast cancer-specific survival (BCSS) and overall survival (OS). Chi-squared tests, Kaplan–Meier method, and Multivariate Cox regression analysis were used for statistical analysis. Propensity score matching (PSM) was performed to minimize the potential selection bias.

**Results:**

A total of 26586 patients were included in this analysis. The median follow-up was 66 months. Of these patients, 15591 (58.6%) patients received RT, RT was recommended but not performed due to patient refusal for 1270 (4.8%) patients, and RT was not recommended for 9725 (36.6%) patients. The 5-year BCSS was 98.3% for patients receiving RT, 97.1% for patients refusal of RT, and 96.4% for patients not recommended RT (P<0.001). The 5-year OS was 88.6% for patients receiving RT, 77.6% for patients who refused RT, and 72.1% for patients not recommended RT (P<0.001). Multivariate Cox regression analyses showed that patients who received adjuvant RT after BCS had significantly better BCSS (hazard ratio [HR] 0.523, 95%confidence interval [CI] 0.447-0.612, P<0.001) and OS (HR 0.589, 95%CI 0.558-0.622, P<0.001) compared to those without RT. A total of 7721 pairs of patients were matched successfully between those with and without RT using PSM. The results also showed that patients who received RT after BCS had significantly better BCSS (HR 562, 95%CI 0.467-0.676, P<0.001) and OS (HR 0.612, 95%CI 0.0.575-0.652, P<0.001) compared to those without RT.

**Conclusions:**

These data suggest that individual counseling is important for treatment decision-making in elderly breast cancer patients with T1-2N0 and ER-positive disease. Given the relatively lower toxicity of modern RT techniques, adjuvant RT should be recommended in patients with high life expectancy.

## Introduction

Breast cancer (BC) is the most common malignancy in women worldwide (1). The probability of BC diagnosis increases with age. The majority of BC occurs in women aged ≥60 years, and 42% of patients were aged ≥70 years at BC diagnosis (2, 3). BC in the elderly is often indolent with positive expression of estrogen receptor (ER) and progesterone receptor (PR) (4). Because patients in the elderly are often excluded from clinical trials, limited data is available for determining the decision on escalation and de-escalation of treatment in this population (5).

Despite an increasing incidence of BC in the elderly, the optimal management of BC in the elderly remains unclear, and the treatment in the elderly mainly refers to their younger counterpart. In clinical practice, treatment decisions for elderly patients with BC are based individually on the assessment of comorbidities, functional status, expected tolerance, and life expectancy (6). In elderly BC patients with T1-2N0 and ER-positive disease, the role of postoperative radiotherapy (RT) in those treated with breast-conserving surgery (BCS) has been controversial in the last two decades.

Several prospective studies have shown that adjuvant RT after BCS may substantially decrease the local recurrence risk, while no benefit of BC-specific death and overall survival (OS) with the addition of RT to BCS among BC patients with aged ≥70 years and ER-positive disease (7, 8). The National Comprehensive Cancer Network guidelines allow for the use of BCS plus endocrine therapy without adjuvant RT in women aged ≥70 years with nodal negative disease and ER-positive, T1 BC (9). However, there were also approximately 60% of patients receive RT after BCS in clinical practice (10). Moreover, although the information on BC death was not analyzed, several population-based studies have shown that the receipt of postoperative RT was associated with a better OS compared to those without RT (11, 12). Therefore, the importance and necessity of RT after BCS in elderly BC remains considerably controversial. In recent years, great progress has been made in the endocrine therapy of ER-positive BC (13). In light of this, our study aimed to investigate whether adjuvant RT after BCS was associated with better survival among elderly (≥70 years) BC patients with T1-2N0 and ER-positive disease in the contemporary model of endocrine therapy.

## Materials and methods

### Patients

**Figure 1:**
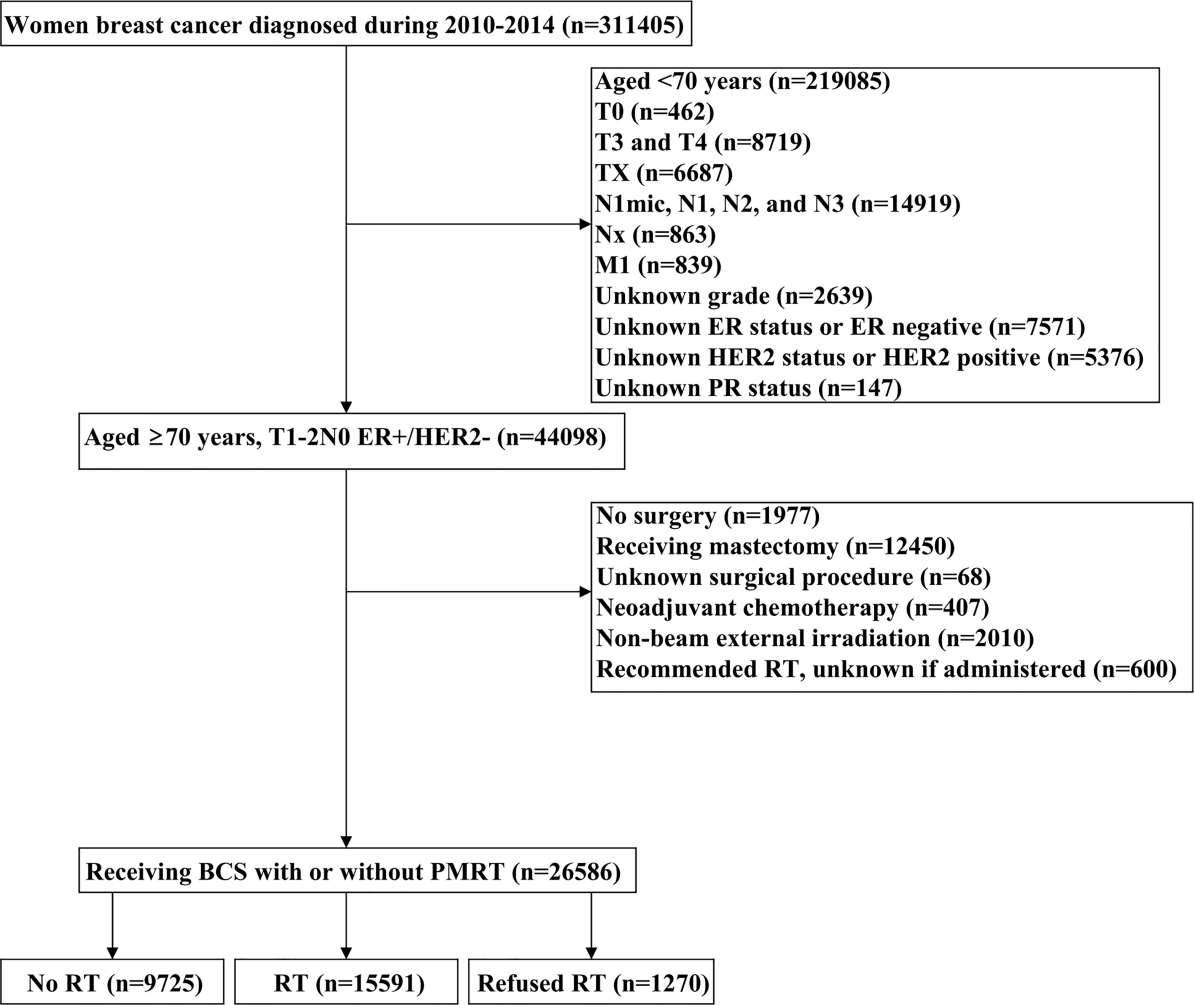
Flow diagram of the study cohort.

Patients diagnosed with BC between 2010 and 2014 were identified from the Surveillance, Epidemiology, and End Results (SEER) program (14). We included patients who met the following criteria: 1) T1-2N0 BC receiving BCS with or without postoperative RT; 2) aged ≥70 years with ER-positive and human epidermal growth factor receptor 2 (HER2) negative; 3) available for histology. The patient selection flowchart has listed in **Figure 1**. Patients who were diagnosed with metastatic disease at BC diagnosis, who received non-beam irradiation, and who received systemic therapy before BCS were excluded. This study used a public de-identified SEER database and the institutional review board approval was waived.

### Measures

We extracted the following data from the SEER program in the analysis: age, race, tumor grade, histology, tumor (T) stage, PR status, chemotherapy use, and RT use. To reduce bias in this retrospective analysis, data were specifically extracted to divide patients into three groups: patients who were recommended to receive RT but ultimately did not receive it due to patients refusal; patients who received adjuvant RT; and patients for whom RT was not recommended and not given. The primary endpoints in the current study were 5-year breast cancer-specific survival (BCSS) and OS. BCSS was defined as the time from the BC diagnosis to the death of BC, and OS was defined as the time from the BC diagnosis to the death related to any cause. The events of locoregional recurrence or distant metastasis were unavailable in the SEER database. Therefore, survival endpoints regarding locoregional recurrence or distant metastasis could not be evaluated in our study.

### Statistical analysis

Descriptive characteristics of patients with and without RT after BCS were compared using Chi-squared tests for categorical variables. A 1:1 propensity score matching (PSM) between those with and without adjuvant RT after BCS was conducted to balance the potential confounders using the following variables: age, race, grade, histology, T stage, PR status, and chemotherapy use. Survival curves were generated using the Kaplan–Meier method and BCSS and OS were compared by the log-rank test. Multivariate Cox regression analyses were performed to analyze the independent factors associated with BCSS and OS. Covariates for adjustment included age at diagnosis, race, histology, tumor grade, T stage, PR status, and chemotherapy use. All statistical analyses were conducted using SPSS software (version 22.0; IBM Corporation, Armonk, NY, USA) or MedCalc Statistical Software version 18.2.1 (MedCalc Software bvba, Ostend, Belgium). All statistical tests were based on 2-sided probability and a P less than 0.05 was considered to be statistically significant.

## Results

**Figure 2:**
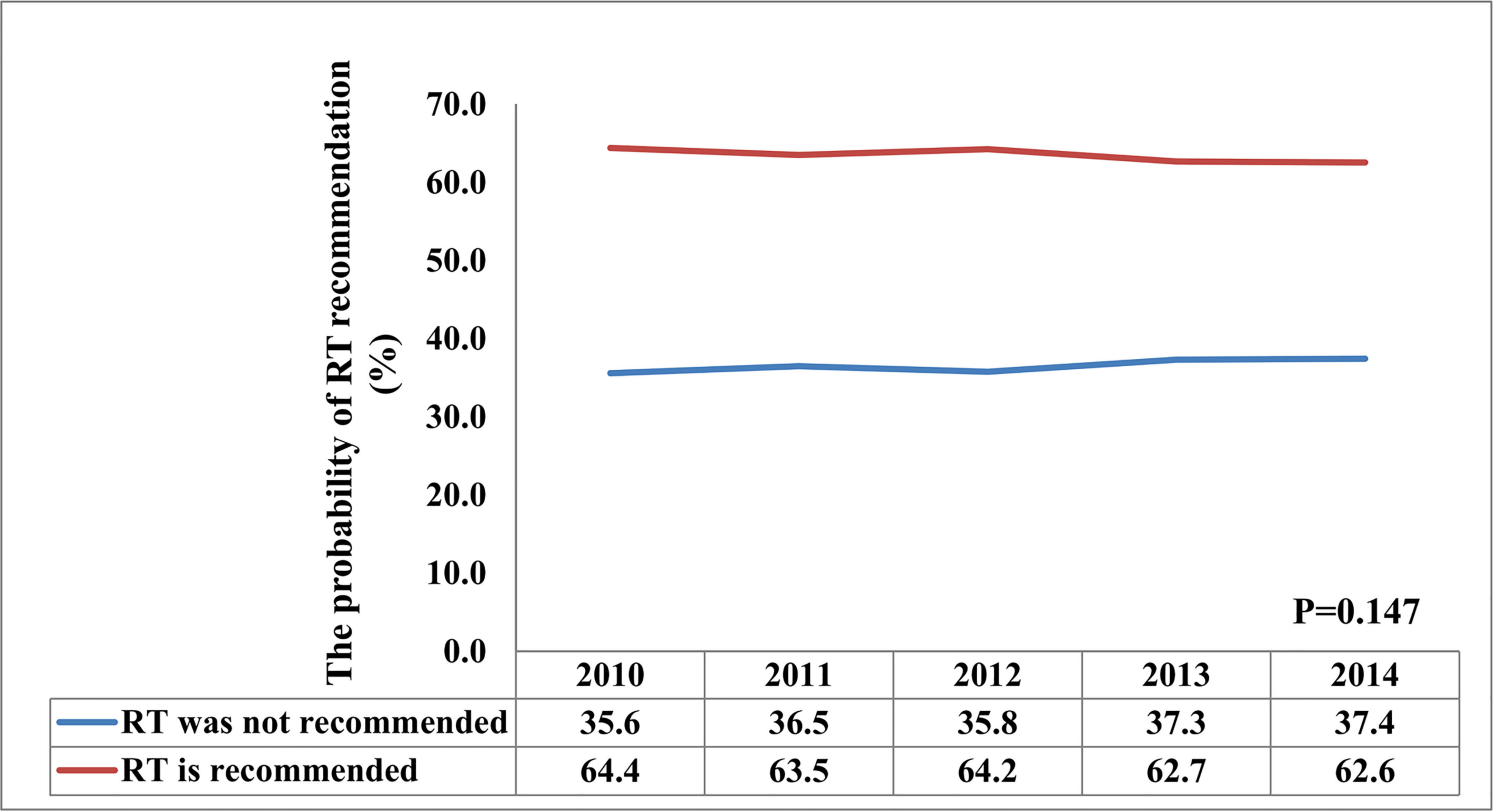
The proportion of patients with and without adjuvant radiotherapy between 2010 and 2014.

A total of 26586 patients who met our cohort definition were included in this study (**Table 1**). Of these patients, 15591 (58.6%) patients received RT, RT was recommended but not performed due to patient refusal for 1270 (4.8%) patients, and RT was not recommended for 9725 (36.6%) patients. There were no significant differences in the pattern of patients’ recommended RT over time (P=0.147) (**Figure 2**). The proportion of patients who did not recommend RT was 35.6% and had a slight increase to 37.4% in 2014, the proportion recommending RT was 64.4% in 2010 and had a slight decrease to 62.6% in 2014.

**Table 1:** Patients’ baseline characteristics before and after propensity score matching.

Variables	Before PSM					After PSM			
	n	No RT (%)	RT (%)	Refused RT (%)	P	n	No RT	RT	P
Age (years)									
70-74	9742	2276 (23.4)	7136 (45.8)	330 (26.0)	<0.001	4510	2255	2255	1
75-79	7545	2321 (23.9)	4889 (31.4)	335 (26.4)		4590	2295	2295	
80-84	5305	2428 (25.0)	2569 (16.5)	308 (24.3)		4390	2195	2195	
≥85	3994	2700 (27.8)	997 (6.4)	297 (23.4)		1952	976	976	
Race									
Non-Hispanic White	21687	7915 (81.4)	12707 (81.5)	1065 (83.9)	0.015	12548	6274	6274	1
Non-Hispanic Black	1627	611 (6.3)	957 (6.1)	59 (4.6)		932	466	466	
Hispanic (All Races)	1692	656 (6.7)	954 (6.1)	82 (6.5)		1062	531	531	
Other	1580	543 (5.6)	973 (6.2)	64 (5.0)		900	450	450	
Histology									
Invasive ductal carcinoma	21483	7834 (80.6)	12608 (80.9)	1041 (82.0)	<0.001	12632	6313	6313	1
Invasive lobular carcinoma	2830	892 (9.2)	1829 (11.7)	109 (8.6)		1408	704	704	
Other	2273	999 (10.3)	1154 (7.4)	120 (9.4)		1402	701	701	
Grade									
Well differentiated	10491	4117 (42.3)	5806 (37.2)	568 (44.7)	<0.001	6456	3228	3228	1
Moderately differentiated	13127	4657 (47.9)	7893 (50.6)	577 (45.4)		7532	3766	3766	
Poorly/undifferentiated	2968	951 (9.8)	1892 (12.1)	125 (9.8)		1454	727	727	
Tumor stage									
T1	22238	8192 (84.2)	12974 (83.2)	1072 (84.4)	0.077	13314	6657	6657	1
T2	4348	1533 (15.8)	2617 (16.8)	198 (15.6)		2128	1064	1064	
PR status									
Negative	3021	1045 (10.7)	1830 (11.7)	146 (11.5)	0.053	1598	799	799	1
Positve	23565	8680 (89.3)	13761 (88.3)	1124 (88.5)		13844	6922	6922	
Chemotherapy									
No	25876	9576 (98.5)	15039 (96.5)	1261 (99.3)	<0.001	15186	7593	7593	1
Yes	710	149 (1.5)	552 (3.5)	9 (0.7)		256	128	128	

**Figure 3:**
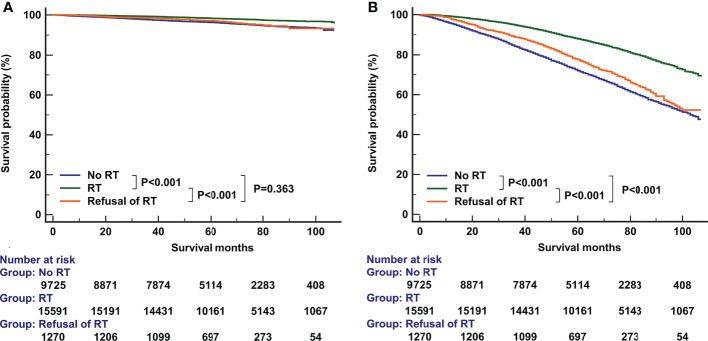
The effect of adjuvant radiotherapy on breast cancer-specific survival **(A)** and overall survival **(B)** before propensity score matching.

The median follow-up time was 66 months (range, 0-107 months). A total of 6551 patients had died, including 766 patients who died with BC. The 5-year BCSS and OS was 97.6% and 81.7%, respectively. The 5-year BCSS was 98.3% for patients receiving RT, 97.1% for patients refusal of RT, and 96.4% for patients not recommended RT (P<0.001, **Figure 3A**). The 5-year OS was 88.6% for patients receiving RT, 77.6% for patients who refused RT, and 72.1% for patients not recommended RT (P<0.001, **Figure 3B**). In univariate analyses, patients who received RT had a better BCSS compared to those who were not recommended RT (hazard ratio [HR] 2.244, 95%confidence interval [CI] 1.934-2.599, P<0.001) as did patients who refused RT (HR 1.946, 95%CI 1.429-2.650, P<0.001). Similar results were found regarding OS (no RT. vs. RT, HR 2.357, 95%CI 2.242-2.479, P<0.001; the refusal of RT vs. RT, HR 1.940, 95%CI 1.742-2.160, P<0.001). The year of diagnosis did not seem to be associated with survival outcomes.

Multivariate Cox survival analyses were performed on the 26586 patients (**Table 2**). Patients who refused RT had comparable BCSS compared to those who did not receive RT (HR 0.890, 95%CI 0.655-1.208, P=0.454). Those who received RT had better BCSS compared to those who did not receive RT (HR 0.523, 95%CI 0.447-0.612, P<0.001). Regarding OS, patients who refused RT (HR 0.868, 95%CI 0.780-0.965, P=0.009) or patients who received RT (HR 0.589, 95%CI 0.558-0.622, P<0.001) had better OS compared to those who did not receive RT. Age at diagnosis, race, tumor grade, T stage, and PR status were also independent prognostic factors associated with survival outcomes.

**Table 2:** Multivariate prognostic analysis before propensity score matching.

Variables	BCSS			OS		
	HR	95%CI	P	HR	95%CI	P
Age (years)						
70-74	1			1		
75-79	1.261	1.023-1.555	0.030	1.559	1.445-1.683	<0.001
80-84	1.737	1.407-2.144	<0.001	2.489	2.310-2.682	<0.001
≥85	2.830	2.298-3.486	<0.001	4.367	4.052-4.706	<0.001
Race						
Non-Hispanic White	1			1		
Non-Hispanic Black	1.035	0.777-1.378	0.814	1.066	0.963-1.179	0.217
Hispanic (All Races)	0.073	0.804-1.432	0.630	0.876	0.786-0.976	0.017
Other	0.769	0.544-1.086	0.136	0.707	0.626-0.798	<0.001
Histology						
Invasive ductal carcinoma	1			1		
Invasive lobular carcinoma	0.965	0.765-1.217	0.763	0.941	0.867-1.020	0.141
Other	0.896	0.681-1.179	0.433	1.045	0.961-1.137	0.303
Grade						
Well differentiated	1			1		
Moderately differentiated	1.437	1.204-1.716	<0.001	1.071	1.016-1.130	0.012
Poorly/undifferentiated	2.922	2.431-3.681	<0.001	1.348	1.246-1.458	<0.001
Tumor stage						
T1	1			1		
T2	2.908	2.500-3.383	<0.001	1.648	1.554-1.747	<0.001
PR status						
Negative	1			1		
Positve	0.647	0.540-0.776	<0.001	0.916	0.852-0.985	0.018
Chemotherapy						
No	1			1		
Yes	1.310	0.923-1.853	0.127	0.863	0.719-1.037	0.116
RT						
No	1			1		
Yes	0.523	0.447-0.612	<0.001	0.589	0.558-0.622	<0.001
Refused	0.890	0.655-1.208	0.454	0.868	0.780-0.965	0.009

**Figure 4:**
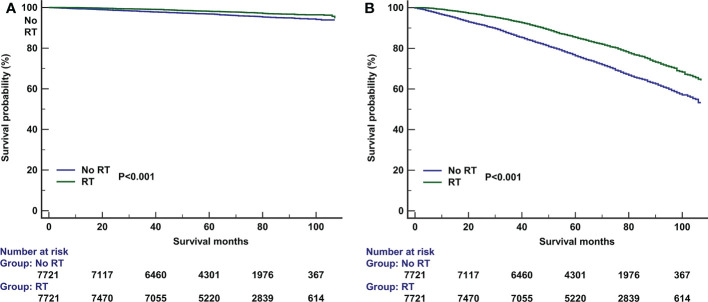
The effect of adjuvant radiotherapy on breast cancer-specific survival **(A)** and overall survival **(B)** after propensity score matching.

PSM was used with the same dataset for the two cohorts of patients with (n=15591) and without (n=9725) adjuvant RT after BCS. Of the 9725 patients without RT, 7721 (79.4%) were matched successfully. Therefore, a total of 7721 pairs of patients were matched successfully (**Table 1**). The 5-year BCSS in those with and without RT was 98.1% and 96.8%, respectively (P<0.001, **Figure 4A**). The 5-year OS in those with and without RT was 85.4% and 76.3%, respectively (P<0.001, **Figure 4B**). The results of multivariate Cox survival analyses also showed that patients who received RT had better BCSS (HR 0.562, 95%CI 0.467-0.676, P<0.001) and OS (HR 0.612, 95%CI 0.575-0.652, P<0.001) compared to those who without RT (**Table 3**).

**Table 3:** Multivariate prognostic analysis after propensity score matching.

Variables	BCSS			OS		
	HR	95%CI	P	HR	95%CI	P
Age (years)						
70-74	1			1		
75-79	1.539	1.536-1.164	0.002	1.600	1.449-1.767	<0.001
80-84	1.859	1.420-2.427	<0.001	2.436	2.218-2.675	<0.001
≥85	3.138	2.363-4.166	<0.001	4.223	3.819-4.669	<0.001
Race						
Non-Hispanic White	1			1		
Non-Hispanic Black	0.966	0.657-1.419	0.859	1.008	0.883-1.152	0.901
Hispanic (All Races)	0.925	0.630-1.359	0.692	0.897	0.785-1.026	0.113
Other	0.589	0.357-0.973	0.039	0.680	0.580-0.796	<0.001
Histology						
Invasive ductal carcinoma	1			1		
Invasive lobular carcinoma	1.184	0.886-1.583	0.253	1.010	0.908-1.122	0.861
Other	0.894	0.620-1.289	0.548	1.072	0.962-1.195	0.209
Grade						
Well differentiated	1			1		
Moderately differentiated	1.298	1.041-1.618	0.020	1.063	0.994-1.137	0.076
Poorly/undifferentiated	2.903	2.227-3.785	<0.001	1.378	1.243-1.527	<0.001
Tumor stage						
T1	1			1		
T2	2.772	2.271-3.385	<0.001	1.581	1.462-1.710	<0.001
PR status						
Negative	1			1		
Positve	0.642	0.506-0.815	<0.001	0.942	0.855-1.036	0.218
Chemotherapy						
No	1			1		
Yes	1.180	0.689-2.018	0.547	0.822	0.619-1.092	0.176
RT						
No	1			1		
Yes	0.562	0.506-0.815	<0.001	0.612	0.575-0.652	<0.001

## Discussion

In this study, using the data from the SEER program, we found that although the prospective studies have shown no survival benefit of RT after BCS in elderly BC patients, there were also approximately 60% of patients receiving RT after BCS in clinical practice. In addition, we also found that the addition of RT to BCS in elderly patients had a better BCSS and OS.

Since 2004, the findings from the GALGB 9343 trial supported that BCS plus endocrine therapy without RT yields similar OS and acceptable locoregional recurrence rate in women aged ≥70 years with T1N0 ER-positive BC compared to those received adjuvant RT after BCS (15). After that, the BCS rate increased over time for early-stage elderly BC (16), whereas the RT rate decreased after BCS over time. A prior SEER study found that approximately 68.6% of patients treated between 2000 and 2004 received RT after BCS, and there were 61.7% of patients received RT after BCS between 2005 and 2009 (P<0.001) (10). In our study, the proportion of RT recommendations after BCS did not have significant differences from 2010 to 2014 (P=0.147), and there were also 64.4% and 62.6% of patients recommended for RT after BCS in the year 2010 and 2014, respectively. Therefore, our findings suggest that the publication of the GALGB 9343 trial resulted in a small but significant decrease in RT delivery in elderly patients. However, the overall use of RT has shown a steady trend in recent years.

In this study, a total of 6551 patients died, while only 11.7% (n=766) of them died from BC. There was approximately 20-50% of patients had comorbidities at the BC diagnosis (12, 17, 18). Therefore, competing mortality is a major factor affecting survival in elderly BC patients. However, several studies have shown that comorbidities, education level, area of residence, receipt of endocrine therapy, and distance to the nearest RT clinic were not associated with the omission of RT after BCS in this population (14, 19). In clinical practice, age, marital status, and tumor grade were the main factors associated with RT decision-making in the elderly (14, 19). In our study, we also found that patients with younger age and higher tumor grade were more likely to receive RT. Several studies also showed that patients with younger age were more likely to receive chemotherapy and long-lasting endocrine therapy (20, 21). Although RT may lead to treatment-related acute and chronic toxicities and increase health care costs (22, 23), several studies have found that the use of RT did not negatively impact the quality of life of elderly patients (8, 24). With the progress of RT techniques and the altered RT fractionation (25, 26), lower RT-induced toxicity and a short course of RT may increase elderly patients’ compliance with RT.

Although approximately two-thirds of the elderly patients still receive RT after BCS in clinical practice, the exact role of RT in this population still needs to be further elucidated. A meta-analysis by Matuschek et al. including 3766 patients from five randomized trials found that the addition of RT to endocrine therapy improved local control but did not have an impact on OS (27). A study from Germany found that adjuvant RT did not improve recurrence-free survival (P=0.651) and OS (P=0.573) (28). However, the small number of patients in the above study may have influenced the statistical results due to the high survival rate and low recurrence rate in this population (n=950). Another study from Italy found that adjuvant RT had no effect on BC death and distant metastasis, and the 15-year local recurrence rate in patients without RT was significantly higher than that in patients with RT (14.6% vs. 0.8%, P= 0.004) (29). However, it should be noted that early patient enrollment, imprecise RT techniques, and insufficient endocrine therapy may impact the survival benefit of RT. Recently, two studies from the National Cancer Database (NCDB) showed a positive impact of RT in this population, they found that RT could improve the OS of patients, especially for those treated with endocrine therapy and RT (11, 12). However, the NCDB does not record BC-related deaths in patients. A large cohort (n=26279) study from the Ontario data also showed the addition of RT was associated with a lower recurrence (P<0.001) and death (P<0.001) even after accounting for age and comorbidities (30). In our study, we found that the addition of RT can not only improve OS but also improve BCSS in this patient subset before and after PSM. The participants in the clinical trial were highly selected and differed from those in routine clinical practice on several factors, including adherence to endocrine treatment, treatment facility and quality, follow-up frequency, etc. Therefore, our study raised the reconsideration for the omission of adjuvant RT after BCS in routine clinical practice.

Our study has OS benefits in those treated with RT, perhaps because we analyzed real-world data from a population-based cohort rather than highly selected cases in clinical trials. That hypothesis might indicate that the OS benefit with the addition of RT is apparent only in the cohort with a high sample size. The difference might also be attributable to the fact that published randomized trials tend to use stricter inclusion criteria and more extensive local surgical resection (31–35). However, the selection bias still could not be ruled out regarding the OS benefit of RT in our study. Despite the growing body of relevant evidence, many unanswered questions remain. Although high-quality evidence exists, the relatively short follow-up time and limited statistical power limit the results of prospective randomized trials for clinical practice. Studies with longer follow-up are required to detect significant differences in OS, but it is worth emphasizing that large retrospective population based-studies including ours have found early differences in OS (11, 12, 30). The large population-based studies not only provided real-world clinical evidence but may also compensate for any retrospective design-related drawbacks.

Several limitations should be acknowledged in our study. First, despite our best efforts to account for potential confounders with PSM, it is impossible to eliminate the risk of selection bias. Second, details regarding endocrine therapy were not recorded in the SEER program. However, the findings from a previous NCDB study included 113505 patients aged >65 years, the proportions of receiving endocrine therapy alone, RT alone, and RT plus endocrine therapy were 20.7%, 16.1%, and 63.2%, respectively (11). The BCSS of the patients in our study was 97.6%, therefore, we could assume that the majority of patients in our study received endocrine therapy. Third, the patterns of locoregional recurrence and distant metastasis were not recorded in the SEER database. Moreover, the median follow-up was only 66 months in this study because the information on HER2 status was not collected in the SEER until 2010, which was insufficient to assess the long-term survival of patients. Finally, age is closely related to comorbidities and frailty. However, comorbidity, frailty, and functional status were not recorded in the SEER database, and could not be evaluated in our study.

## Conclusions

In conclusion, our findings suggest that individual counseling is important for treatment decision-making in elderly patients with early-stage BC. Given the relatively lower toxicity of modern RT techniques, adjuvant RT should be recommended in patients with high life expectancy. However, given that prospective clinical trials have failed to show an OS benefit with the addition of RT in this population, we caution using the retrospective data to draw a conclusion on OS. More studies are required to determine the optimal candidates for RT recommendation in this population.

## Data availability statement

Publicly available datasets were analyzed in this study. We have got the permission to access the SEER database on purpose of research only (Reference number: 11025-Nov2016). This data can be found here: www.seer.cancer.gov.

## Ethics statement

Ethical review and approval was not required for the study on human participants in accordance with the local legislation and institutional requirements. Written informed consent for participation was not required for this study in accordance with the national legislation and the institutional requirements.

## Author contributions

S-PY, PZ, and C-LL drafted the manuscript. S-GW acquired the datasets. S-GW and Z-YH conceived the study. S-GW conducted the statistical analyses. S-GW and S-PY participated in the study design. All authors contributed to the article and approved the submitted version.

## Funding

This work was partly supported by the Social Development Projects of Key R & D Programs in Hainan Province (No. ZDYF2022SHFZ130), Commission Young and Middle-aged Talents Training Project of Fujian Health Commission (No. 2019-ZQNB-25), the Natural Science Foundation of Fujian Province (No. 2020J011240), and Natural Science Foundation of Hainan Province (No. 819QN345).

## Acknowledgments

The authors acknowledge the efforts of the Surveillance, Epidemiology, and End Results (SEER) Program tumor registries in the creation of the SEER database.

## Conflict of interest

The authors declare that the research was conducted in the absence of any commercial or financial relationships that could be construed as a potential conflict of interest.

## Publisher’s note

All claims expressed in this article are solely those of the authors and do not necessarily represent those of their affiliated organizations, or those of the publisher, the editors and the reviewers. Any product that may be evaluated in this article, or claim that may be made by its manufacturer, is not guaranteed or endorsed by the publisher.
